# Does Self-Selection Affect Samples’ Representativeness in Online Surveys? An Investigation in Online Video Game Research

**DOI:** 10.2196/jmir.2759

**Published:** 2014-07-07

**Authors:** Yasser Khazaal, Mathias van Singer, Anne Chatton, Sophia Achab, Daniele Zullino, Stephane Rothen, Riaz Khan, Joel Billieux, Gabriel Thorens

**Affiliations:** ^1^Geneva University HospitalsGenevaSwitzerland; ^2^Geneva UniversityGenevaSwitzerland; ^3^Laboratory for Experimental Psychopathology, Psychological Science Research Institute, Catholic University of LouvainLouvain-La-NeuveBelgium

**Keywords:** Internet, bias, online survey, self-selection, random sample, World of Warcraft, massively multiplayer online role-playing

## Abstract

**Background:**

The number of medical studies performed through online surveys has increased dramatically in recent years. Despite their numerous advantages (eg, sample size, facilitated access to individuals presenting stigmatizing issues), selection bias may exist in online surveys. However, evidence on the representativeness of self-selected samples in online studies is patchy.

**Objective:**

Our objective was to explore the representativeness of a self-selected sample of online gamers using online players’ virtual characters (avatars).

**Methods:**

All avatars belonged to individuals playing World of Warcraft (WoW), currently the most widely used online game. Avatars’ characteristics were defined using various games’ scores, reported on the WoW’s official website, and two self-selected samples from previous studies were compared with a randomly selected sample of avatars.

**Results:**

We used scores linked to 1240 avatars (762 from the self-selected samples and 478 from the random sample). The two self-selected samples of avatars had higher scores on most of the assessed variables (except for guild membership and exploration). Furthermore, some guilds were overrepresented in the self-selected samples.

**Conclusions:**

Our results suggest that more proficient players or players more involved in the game may be more likely to participate in online surveys. Caution is needed in the interpretation of studies based on online surveys that used a self-selection recruitment procedure. Epidemiological evidence on the reduced representativeness of sample of online surveys is warranted.

## Introduction

An increasing number of medical and psychological studies are performed through online surveys. Compared with face-to-face interviews, Internet-based surveys can quickly reach more potential participants, reduce measurement error and bias related to answers on stigmatizing topics, and enhance the inclusion of least represented or “quasi-secret” and stigmatized population groups that are usually difficult to reach and recruit [1-5]. Costs can be more easily contained with Internet-based surveys, and data collection can be simpler and more reliable compared to traditional paper-and-pencil data entry procedures. Some studies suggest that the quality of the data provided by Internet-based surveys is at least as good as in those collected by traditional paper-and-pencil methods on self-selected samples [6-8].

Some Web surveys have been based on the assessment of a whole population or on samples obtained using random sampling procedures (ie, a sampling technique whereby all individuals in the population have an equal chance of being selected, eg, emailing a random sample of students in a university). For instance, Internet-based surveys among students enrolled by email have generated valid and reliable estimations of substance use [3,9,10], comparable to those obtained in studies that applied ordinary mail invitation letters or phone calls to recruit participants.

Many Web studies are, however, self-selection surveys [11] that are not based on probability sampling [12], particularly in health-related studies. Websites and online social networks such as Facebook appeared to be a viable recruitment option for the assessment of health behaviors [13,14]. However, lack of researchers’ knowledge about the website members’ contacts leads to the impossibility of obtaining a random sampling. The survey questionnaire is then usually put on the Web. Potential participants are among those people with Internet access who visit the website, find the study information, and decide to complete the survey. In the case of self-selection surveys, the researcher then has no control over the selection process and can work only on the design of the study advertisement (such as graphics and content, length of questions, possible incentives) or on a selection of an appropriate website or forum to promote the response rate to electronic questionnaires [15]. Online self-selection surveys are thus particularly subject to coverage and selection bias, which undermines the external validity of studies and the interpretation of findings [12].

Coverage bias is possibly influenced by patterns related to Internet access or to specific website access (ie, differences between people with or without Internet access) and to the possibility of being particularly interested in the study for reasons that may or may not be related to the content and/or objective of the survey itself. Furthermore, exposure to the advertisement is influenced by the time spent on a specific website, and chain sampling bias may also occur because heavy users may be more prone to share information about the study with other contacts than light users [16].

Self-selection bias (individuals who select themselves for the survey) may be of great importance [12], notably, in consideration of the usually relatively low participation rate [16]. It is difficult to estimate the impact of any selection bias because information on non-participants is usually not available, and comparisons between the included and the excluded samples are not feasible [12].

There is some evidence that the self-selected samples of Internet-based surveys may systematically differ from samples drawn from the general population using other sampling procedures [16,17]. One study showed that an Internet-based study sample had higher past month rates of alcohol and marijuana use than those found in other similar and non-self-selected samples of smokers (behaviors also possibly more easy to disclose online) [17]. Similarly, comparison of registry-recruited cancer survivors with an online recruited sample found that the Internet sample has lower social support and greater mood disturbances than the cancer-based registry-recruited one [18]. Another study found also that participants who preferred online surveys to paper-pencil questionnaires differed from their counterparts on a number of sociodemographic variables [19]. The effect of the self-selection bias is also possibly important in large sample size studies, as suggested from differences in actual election results and a number of online opinion surveys [12].

To the best of our knowledge, studies are lacking on the possible differences between “pure or perfect” random samples and self-selected samples of users of specific Internet services such as online games or social network websites. This weakness is possibly explained by the difficulty for researchers, independently from websites owners, to obtain random samples of Internet users on specific websites.

The online game World of Warcraft (WoW) offers some possibilities to approach the question of selection and self-selection bias in online surveys. WoW gamers have been specifically studied in online self-selected surveys in attempts to assess motivations to play and possible psychological factors associated with gaming addiction [20-23].

In WoW, players assume the role of a fictional character, or “avatar”. An avatar is characterized by a number of elements such as name and visual representation. The avatar’s progression is a core attribute of WoW, implying that an avatar will develop new skills and powers as rewards for the success obtained during in-game missions or quests (eg, beating a monster, finding something specific, exploring areas of the game). Each avatar’s progression is accessible via the “Armory”, an official database reporting the achievements related to each avatar evolving in WoW [24]. Players commonly regroup themselves in guilds (hierarchical organizations of avatars with shared objectives and backgrounds). Each guild has its particular regulations. Players who want to join a given guild usually need to contact the guild’s chief and explain their motivations to join the guild and to give some evidence that their avatar meets the guild’s conditions [22].

Furthermore, the psychological characteristics of the gamers, such as motivation to play [21,23,25], have been shown to be associated with actual in-game behaviors and achievements as reported by the Armory scores [22]. Accordingly, the Armory scores of a given avatar, to some extent, reflect the game style and commitment of a given WoW player (ie, details of the achievements reached). The variables assessed in the present study were extracted from the armory and could be considered as an “ecological measure” (the measures automatically collected during game play represent direct in-game behaviors) of both the commitment of the players and their playing preferences [22].

WoW thus offers the possibility of comparing characteristics of the progression of self-selected avatars to a “pure or perfect” random sample of avatars. A sample is considered pure or perfect in the sense that every randomly selected avatar is actually included in the sample, whereas in classic studies (ie, non-self-selected samples), subjects are allowed to refuse to participate, which could induce a selection bias.

The aim of the current study was to compare the armory characteristics of two self-selected samples with a random sample of WoW avatars.

## Methods

### Summary

The study compared a random sample of avatars with two different self-selected samples. Only the avatars at the maximum level of the game version at inclusion were included in the study. The mechanics of “leveling” is as follows: When a player starts to play with an avatar, this avatar automatically starts at level 1. While playing, avatars gain experience points and these points allow the avatar to reach new levels (10,000 points to reach level 2; 25,000 to reach level 3, etc). Each new game version allows avatars to gain higher maximum levels. At the creation of the game, the maximum level an avatar could reach was 60. Each time that an expansion pack is released, the highest reachable level is raised (80 for Wrath of the Lich King and 85 for the Cataclysm versions of the game).

As described below, most of the avatars of the self-selected samples were at the maximum level. This level is not the maximum of the possible in-game achievements (reflected by the armory scores) but something like a mandatory “pass” for certain important tasks in the game (especially raids). To be considered as a seriously involved player, an avatar has to reach this maximum. So, including only avatars at this maximum level makes the avatars more comparable in terms of game “involvement”.

### First Self-Selected Sample

The first self-selected sample (self-selected sample 1) of avatars was from a study on the relationships between players’ self-reported motives to play and their in-game behaviors [21]. The study was performed between June and December 2010 and was approved by the ethical committee of the Psychology Department of the University of Geneva. Inclusion criteria were French-speaking WoW players who were aged 18 years or older. Participants were recruited through advertisements posted in dedicated French-language forums: a guilds forum, an official Blizzard WoW forum, and more general online and video games forums. Some participants also joined the study after having heard about it in the local press or from television interviews. All participants gave online consent prior to starting the online survey. So, the sample included the avatars of online gamers who actively participated in the study given the identity of their avatars. Concomitant avatars’ in-game behaviors were collected through the French Armory website [24].

**Figure 1:**
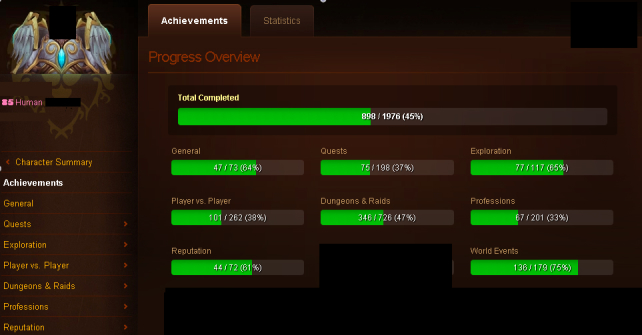
Example from the Armory.

The WoW avatar achievements studied here and reported in the Armory (Figure 1) are as follows: general achievements, quests (progression in the various available quests in the game), exploration (exploring each area in the game), player versus player (fighting other players), dungeons and raids (raids or dungeon crawling, ie, specific missions needing a group of players to achieve a common objective), profession, reputation, word events, and total completed. These achievement scores (Figure 1) were reported in the following format: score, maximum score (maximum possible score), and percentage of the maximum score. This percentage is calculated as the ratio of the scores gained by the player to the maximum possible score of the achievement in question multiplied by 100. For instance, a player who has obtained a score of 20 for quests out of a maximum of 127 is credited with a percentage of 15.75. All other percentages considered for the analysis were also calculated in the same way, and their means were compared across samples. Taking the percentage allows comparison of the avatars in different time periods despite possible modification in the WoW game. Some achievements such as “feats of strengths” and “total points” were not expressed as a percentage and were therefore not included in the study because of the difficulty in interpreting these scores in the case of game modifications.

Of a total of 1601 participants who started the survey (self-selection), 690 completed it (43.10%) and concomitantly provided the names of their avatar and the realms in which they play (ie, the name of their server, which is necessary to identify the avatar). Among these avatars, only those with a level of 80 were included in the present study, which represents 663 avatars of 690 (96.1%). This level was the higher one at the time of inclusion of the self-selected sample.

### Second Self-Selected Sample

The second self-selected sample (self-selected sample 2) of avatars was from a study performed between December 2011 and April 2012. Similarly, the sample included the avatars of online gamers who actively participated in the study given the identity of their avatars. Concomitant avatars’ in-game behaviors were collected in the same way as for the first self-selected sample.

The study, approved by the ethical committee of the Psychology Department of the University of Geneva, had similar purposes to the first study and similar recruitment procedures. The sample was assessed with the same measures from the Armory. One important added value of this smaller sample was the time of recruitment, which took place after the release of the Cataclysm version of the game in December 2010; it is therefore a version of the game similar to the one related to the random sample described in the next section. At that time, the maximum level was raised from 80 to 85.

Furthermore, response bias is considered as an “individual” characteristic of a given sample; replication of the results on different samples could be considered as a way to increase the validity of a given study conclusion [26].

In total, 104 participants participated in the survey; of these, 99 avatars (95.2%) had a level of 85 (the maximum) and were included in the present study.

### Random Sample of Avatars

The list of avatars was found on a public website of game players [27] on February 25, 2012. On this website [27], each server was presented with numbered avatars.

Due to the fact that the two self-selected samples were recruited among French speaking WoW players, only the French-speaking population of avatars (found on French servers) was considered in order to ensure group comparability.

Given that most of the avatars of the two self-selected samples (96.1% of the first one, 637/663 avatars, and 95.2% of the second one, 94/99 avatars) were at the maximum level of the game version at the time of recruitment, only those avatars were included in our study and compared with a random sample of avatars. For the sake of comparability, all avatars included in the study were at the 85 level, which is the maximum level related to the Cataclysm version.

To form the random sample, 600 avatars were randomly drawn. The number of selected avatars from each server was proportional to the contribution of each server to the total population. The random allocation was made using a specialized website [28]. The avatars selected by this procedure were then searched for and assessed in the WoW official comprehensive database [24]. Only avatars considered as still active by the WoW game were registered in this database.

### Statistical Analyses

Statistical analyses were performed using SPSS, version 18.0. An initial exploratory analysis involved the calculation of percentages, as well as means and standard deviations of the above-mentioned outcome measures.

To address the research question, analysis of variance (ANOVA) or *t* tests are appropriate in comparing mean percentages across groups. However, although *F* tests are robust against departure from normality, the homogeneity of variance is a strong assumption that must be satisfied for the ANOVA results to be reliable. As percentages and proportions variables are not likely to meet normality and homogeneity of variance assumptions, the arcsine-transformation is often used with this type of data and serves the purpose of normalizing them and stabilizing their variance. First, all variables were expressed as proportions, that is, between 0 and 1, and then they were transformed according to the following formula: y=2arcsin (SQRT(p)), where p stands for proportion.The combined effect of the square root with the inverse sine compresses the upper tail of the distribution and stretches out both tails relative to the middle. The ANOVAs and *t* tests were done on the transformed variables after visual inspections of their normality and Q-Q plots excluded inacceptable patterns. But for the sake of completeness, the tables also display the variables in their original scale. In a first step, the three samples were compared (self-selected sample 1 vs self-selected sample 2 vs random sample) using a one-way between-group ANOVA to explore the impact of each sample on a list of 10 selected variables. In a second step, the two self-selected samples were merged, since they did not differ (shown by post hoc comparison tests), and two-sample *t* tests were done, comparing the random sample with the merged self-selected samples. To account for multiple comparisons testing (10 multiple ANOVAs and *t* tests), we performed Bonferroni corrections deflating alpha type I error so that the adjusted significance level is alpha/10 (here .005). Finally, a chi-square test was carried out to compare proportions of avatar per guild between the two types of samples.

## Results

Table 1 shows the ANOVA results for each variable of interest. Except for “guild membership”, overall, statistically significant differences at the .005 level between the three samples were found for all assessed variables. Bonferroni post hoc tests showed that these differences mainly occurred within each self-selected sample compared with the random sample. However, these differences were not statistically significant between the self-selected sample 1 and the random sample for exploration (*P*=.6), between the self-selected sample 2 and the random sample for total completed (*P*=.1), and between neither one of the self-selected samples and the random sample for guild (*P*=1.0 respectively).

**Table 1:** Comparison of mean values between three samples: self-selected sample 1 (n=663), self-selected sample 2 (n=99), and random sample (n=478) by one-way ANOVA performed on the transformed variables.

WoW characteristics	Variables successively presented in their original and transformed values	Self-selected sample 1, mean (SD)	Self-selected sample 2, mean (SD)	Random sample, mean (SD)	*P* value
Dungeons and raids	Original mean proportion	0.40 (0.20)	0.42 (0.24)	0.28 (0.18)	
	Arcsine-transformed value	1.36 (0.44)	1.38 (0.54)	1.07 (0.43)	<.001
Word events	Original mean proportion	0.40 (0.33)	0.43 (0.34)	0.21 (0.25)	
	Arcsine-transformed value	1.34 (0.81)	1.38 (0.83)	0.85 (0.61)	<.001
Exploration	Original mean proportion	0.62 (0.35)	0.76 (0.26)	0.64 (0.27)	
	Arcsine-transformed value	1.91 (0.85)	2.23 (0.66)	1.91 (0.67)	<.001
General	Original mean proportion	0.60 (0.18)	0.60 (0.18)	0.48 (0.15)	
	Arcsine-transformed value	1.81 (0.44)	1.81 (0.44)	1.54 (0.32)	<.001
Player versus player	Original mean proportion	0.35 (0.19)	0.27 (0.18)	0.20 (0.14)	
	Arcsine-transformed value	1.24 (0.42)	1.04 (0.44)	0.88 (0.37)	<.001
Profession	Original mean proportion	0.48 (0.29)	0.42 (0.28)	0.27 (0.23)	
	Arcsine-transformed value	1.54 (0.67)	1.40 (0.68)	1.02 (0.56)	<.001
Quests	Original mean proportion	0.50 (0.25)	0.40 (0.27)	0.26 (0.19)	
	Arcsine-transformed value	1.61 (0.61)	1.38 (0.69)	1.04 (0.43)	<.001
Reputation	Original mean proportion	0.35 (0.25)	0.39 (0.30)	0.23 (0.22)	
	Arcsine-transformed value	1.24 (0.60)	1.33 (0.75)	0.94 (0.57)	<.001
Total completed	Original mean proportion	0.43 (0.19)	0.54 (0.27)	0.37 (0.21)	
	Arcsine-transformed value	1.42 (0.42)	1.73 (0.70)	1.31 (0.50)	<.001
Involvement in guilds	Original mean proportion	0.87 (0.34)	0.86 (0.35)	0.88 (0.32)	
	Arcsine-transformed value	2.73 (1.06)	2.70 (1.10)	2.77 (1.01)	.7

Comparing the two self-selected samples, Bonferroni’s post hoc tests showed that they differed on the following variables: exploration, player versus player, and quests (*P*<.001 respectively). No further difference was observed for the other variables. We merged these two self-selected samples into one bigger sample, which in turn was compared to the random sample. Table 2 shows the two-sample *t* tests between the new self-selected sample and the random sample. Both samples differed significantly at the .005 level on each variable, except for guild membership and exploration, with the random sample having a similar mean to that of the self-selected sample.

**Table 2:** Comparison of mean values between the merged self-selected sample (n=762) and the random sample (n=478) by *t* test performed on the transformed variables.

WoW characteristics	Variables are successively presented in their original and transformed values	All self-selected sample players, mean (SD)	Random sample, mean (SD)	Mean difference (99.5% CI)	*P* value
Dungeons and raids	Original mean proportion	0.40 (0.20)	0.28 (0.18)		
	Arcsine-transformed value	1.36 (0.45)	1.07 (0.43)	0.29 (0.22-0.36)	<.001
Word events	Original mean proportion	0.40 (0.33)	0.21 (0.25)		
	Arcsine-transformed value	1.34 (0.81)	0.85 (0.61)	0.49 (0.38-0.60)	<.001
Exploration	Original mean proportion	0.64 (0.34)	0.64 (0.27)		
	Arcsine-transformed value	1.95 (0.83)	1.91 (0.67)	0.05 (-0.08 to 0.17)	.3
General	Original mean proportion	0.60 (0.18)	0.48 (0.15)		
	Arcsine-transformed value	1.81 (0.44)	1.54 (0.32)	0.27 (0.21-0.33)	<.001
Player versus player	Original mean proportion	0.34 (0.19)	0.20 (0.14)		
	Arcsine-transformed value	1.21 (0.43)	0.88 (0.37)	0.33 (0.26-0.39)	<.001
Profession	Original mean proportion	0.48 (0.29)	0.27 (0.23)		
	Arcsine-transformed value	1.52 (0.67)	1.02 (0.56)	0.50 (0.40-0.60)	<.001
Quests	Original mean proportion	0.49 (0.25)	0.26 (0.19)		
	Arcsine-transformed value	1.58 (0.62)	1.04 (0.43)	0.55 (0.46-0.63)	<.001
Reputation	Original mean proportion	0.36 (0.26)	0.23 (0.22)		
	Arcsine-transformed value	1.26 (0.63)	0.93 (0.57)	0.32 (0.23-0.42)	<.001
Total completed	Original mean proportion	0.44 (0.21)	0.37 (0.21)		
	Arcsine-transformed value	1.46 (0.48	1.31 (0.50)	0.15 (0.07-0.22)	<.001
Involvement in guilds	Original mean proportion	0.87 (0.34)	0.88 (0.32)		
	Arcsine-transformed value	2.73 (1.06)	2.77 (1.01)	-0.04 (-0.21 to 0.12)	.5

Some guilds were overrepresented in the self-selected samples (a form of guild effect: people from the same guild may encourage their partners to participate in the study). Table 3 shows the number of avatars per guild. The sample sizes here are lower since not every avatar belongs to a guild. The range is between 1 and 11, with one guild from the self-selected sample having 11 participating avatars. A chi-square test reveals that the distribution of avatar per guild is different between the two types of samples.

**Table 3:** Observed (expected**)** number of avatars per guild: random sample (n=421) and self-selected samples (n=662).

Number of avatars per guild	Random sample^a^	All self-selected sample	χ^2^_2_	*P* value
1	363 (345.0)	393 (411.0)		
2	29^b^ (35.6)	49 (42.4)		
3-11	0 (11.9)	26 (14.1)	25.8	<.001

^a^Number of avatars with a guild affiliation.

^b^29 guilds with 2 avatars per guild.

## Discussion

### Principal Findings

In the French-speaking community of WoW players, three samples of avatars, one purely random and two self-selected, were used to assess the potential self-selection bias of Internet-based studies. To our knowledge, this is the first study to include a perfect random sample, since all randomly selected subjects (avatars) were incorporated in the sample.

The method used in this paper is somewhat new, dealing with the opportunity given by the development of online avatars of Internet users. Table 4 gives some details about the similarities and the differences related to Internet surveys, surveys on online gamers, and studies on avatars.

**Table 4:** Comparison of Internet surveys, online game surveys, and studies on avatars.

	Internet survey	Survey on online gamers	Study on avatars from a self-selected sample	Study on avatars from a random sample
Included	Internet users	On-line game users	Fictive character (video game players’ avatar) linked to a given user who self-selected for study participation (as in the two self-selected samples of our study)	Video game players’ avatar selected randomly from a database including all characters (like the random sample of our study)
Active participation in the survey	Mandatory for study participation	Mandatory for study participation	The characteristics of a given avatar are drawn from the Web. Active participation is linked to the fact that the user decided to disclose their avatar’s information for the study.	No possible active participation to a survey. The characteristics of a given avatar are drawn from the Web (like the achievements characteristics of the random sample).
Possible data obtained	Data on the participant (human) Internet user profile (eg, psychological measures, reported Internet use)	Data on the participant (human) Internet-game user (eg, psychological measures, reported Internet use)	Data automatically collected related to the avatars (eg, achievements) or data chosen by the player (eg, avatar name, gender, guild affiliation) like in our study for the self-selected samples. It remains possible to link the characteristics of the avatar with the user who self-selected him or herself (not available in our study).	Data automatically collected related to the avatars (eg, achievements) or data chosen by the player (eg guild affiliation) like in our study for the random sample.
Self-selection bias	Possible self-selection bias. The participant (human) is or is not informed about the survey and decides to or not to participate and to complete the survey.	Possible self-selection bias. The participant (human) is or is not informed about the survey and decides to or not to participate and to complete the survey.	The human user decided to include their avatar in a study (self-selection bias, like for the 2 self-selected samples of our study)	The avatar is selected randomly from a database (like for the random sample of our study).

The samples were compared on the basis of the in-game achievements of the avatars expressed in percentages. This allows comparison of avatars despite possible game modifications, as shown by the lack of differences between the two self-selected samples recruited at two different times. The second self-selected sample and the random sample were both included during the same Cataclysm version of the game, whereas the first self-selected sample was included before this game version.

According to the hypothesis of a self-selection bias, it appears from the study results that a self-selected sample of website users differs from a “pure or perfect” random sample. The self-selected samples had higher scores than the random sample on most of the assessed in-game behavior variables. The self-selected samples appear to be more involved in the game than the random sample avatars. This could occur for different reasons.

To self-select, a player needs first to see the advertisement for a study (eg, “We are looking for active World of Warcraft players, >18 years old, to participate in an online survey on your motives to play and your psychological profile. We will ask the name of your main avatar and match your answers to Blizzard armory’s data. The questionnaire will take approximately 15 minutes of your time”). Second, a player needs to consider participating and to agree to it. Therefore, having the will to be involved in a study could lead to the selection of specific subjects with certain characteristics (eg, personality, game involvement, special interest in the purpose of the study). Because the participants responded to an Internet advertisement for the study, highly involved players are more likely to see the ad than occasional players because of the time spent on WoW-related websites. On the other hand, one could also assume that the will to participate is related to both involvement in the game and an interest in the proposed studies.

The study finding is consistent with those of other studies linking survey participation with involvement (greater interest, connection, and concern related to the given behavior or possibly to the study results) in the assessed behaviors [19,29].

One may hypothesize that the statistically significant results of the study were due to the large sample size and that type I error (finding a difference when in fact there is none) could not be ruled out. But the magnitude of the differences found between the groups (as displayed in Table 2) cannot be imputed to chance alone and therefore does not support this point of view.

### Limitations

Some limitations warrant further consideration. First, the specificity of WoW, including the guild effect (players organized in a guild), may increase the inclusion of participants who are highly involved in the game via a chain sampling bias. This may limit the generalizability of the results to other domains of Internet use–related behaviors. However, most Internet-related activities involve some form of social networking that promotes chain sampling activities and a possibly similar bias.

Second, the study was done on avatars and not directly on people. People may have more than one avatar on WoW. Thus, we cannot exclude the possibility that the results may be partly explained by differences between the avatar chosen by the self-selected sample as representative and the randomly selected avatars. Inclusion of active avatars at the maximum level related to each game extension and particularly lack of statistically significant differences in guild involvement and exploration (for the first self-selected sample) and on total completed (for the second self-selected sample) suggest, however, that the random sample is composed of at least reasonably credible avatars involved in the game.

Although most of the avatars were affiliated with guilds, affiliation was not an inclusion criterion. However, it was used to assess a form of guild effect (ie, higher proportions of avatars from the same guilds in the self-selected groups in comparison to the random one). Furthermore, guild participation could be considered as a useful index of “serious” avatar in-game activity (ie, the avatar was accepted by a guild).

### Conclusions

Because of the important differences between the self-selected samples and the randomly selected sample, and despite the acknowledged limitations, the study invites careful consideration of the conclusions made from online self-selected samples and the possibility of an overrepresentation of subgroups of more involved or more concerned users.

Therefore, it does not appear possible to draw general epidemiological conclusions from Internet-based self-selection surveys (eg, on the prevalence of game addiction among website users or the general population). However, the studies may be of high interest to subgroups of users who are more involved in the game and the study purpose. In particular, such studies may allow the linking together of different assessed variables (such as mood, motives, or personality and a given behavior) in the studied sample. This remains important, particularly because of the possible advantages of online studies (eg, large sample sizes, possible access to people who are usually more difficult to reach, access to stigmatized behaviors).

The possible collaboration with webmasters may further improve understanding of the representativeness of self-selected samples by the random selection of the users (ie, contacting users by email to build a random sample as control group) or by comparison of the responders to non-responders regarding general characteristics such as features related to website use or, to some extent, potential biases regarding clinical variables (eg, game addiction).

## Abbreviations

ANOVA: analysis of variance
CI: confidence interval
WoW: World of Warcraft
